# Monte Carlo dose verification of prostate patients treated with simultaneous integrated boost intensity modulated radiation therapy

**DOI:** 10.1186/1748-717X-4-18

**Published:** 2009-06-15

**Authors:** Nesrin Dogan, Ivaylo Mihaylov, Yan Wu, Paul J Keall, Jeffrey V Siebers, Michael P Hagan

**Affiliations:** 1 Virginia Commonwealth University Medical Center, Radiation Oncology Department, 401 College Street, Richmond, Virginia 23298, USA; 2 Department of Radiation Oncology, University of Arkansas for Medical Sciences, 4301 W. Markham Street, Little Rock, Arizona 72205, USA; 3 Department of Radiation Oncology, Stanford University Cancer Center, 875 Blake Wilbur Drive, Stanford, California 94305, USA

## Abstract

**Background:**

To evaluate the dosimetric differences between Superposition/Convolution (SC) and Monte Carlo (MC) calculated dose distributions for simultaneous integrated boost (SIB) prostate cancer intensity modulated radiotherapy (IMRT) compared to experimental (film) measurements and the implications for clinical treatments.

**Methods:**

Twenty-two prostate patients treated with an in-house SIB-IMRT protocol were selected. SC-based plans used for treatment were re-evaluated with EGS4-based MC calculations for treatment verification. Accuracy was evaluated with-respect-to film-based dosimetry. Comparisons used gamma (γ)-index, distance-to-agreement (DTA), and superimposed dose distributions. The treatment plans were also compared based on dose-volume indices and 3-D γ index for targets and critical structures.

**Results:**

Flat-phantom comparisons demonstrated that the MC algorithm predicted measurements better than the SC algorithm. The average PTV_prostate _D_98 _agreement between SC and MC was 1.2% ± 1.1. For rectum, the average differences in SC and MC calculated D_50 _ranged from -3.6% to 3.4%. For small bowel, there were up to 30.2% ± 40.7 (range: 0.2%, 115%) differences between SC and MC calculated average D_50 _index. For femurs, the differences in average D_50 _reached up to 8.6% ± 3.6 (range: 1.2%, 14.5%). For PTV_prostate _and PTV_nodes_, the average gamma scores were >95.0%.

**Conclusion:**

MC agrees better with film measurements than SC. Although, on average, SC-calculated doses agreed with MC calculations within the targets within 2%, there were deviations up to 5% for some patient's treatment plans. For some patients, the magnitude of such deviations might decrease the intended target dose levels that are required for the treatment protocol, placing the patients in different dose levels that do not satisfy the protocol dose requirements.

## Background

High-dose calculation accuracy and beam delivery is very important for Intensity Modulated Radiotherapy (IMRT). IMRT is typically delivered through a sequence of small fields or with a dynamically moving aperture and sharper dose gradients near boundaries are very common in IMRT fields [1-3]. Most IMRT systems utilize simple and fast dose-calculation algorithms, such as the pencil beam method, during the optimization process. In many systems, a more accurate algorithm, such as the Superposition/Convolution (SC) method, is used for the final dose calculation after leaf sequencing process. However, even relatively sophisticated semi-analytical dose-calculation algorithms such as SC method can be inaccurate for small fields (>3%), especially in regions of dose gradients, in regions of tissue heterogeneities, and for the estimation of multileaf collimator (MLC) leakage [4-6]. Furthermore, treatment fields for simultaneous integrated boost (SIB) IMRT techniques often have larger intensity variations which result in complex MLC patterns and present challenges to dose calculations algorithms because of the effects of radiation transmitted through and scattered from the MLC [7]. For such fields, assumptions used in conventional dose calculation algorithms may break down, causing large dose prediction errors[8,9]. In addition to the dose calculation algorithm type, the major factors of influencing dose calculation accuracy are the beam modeling and the user specific commissioning and tuning of the dose calculation model to match IMRT dose distributions for a particular accelerator.

It has been shown that the use of an algorithm such as Monte Carlo (MC) that can explicitly account for MLC leakage and scatter can provide more improved dose calculation accuracy when compared to measurements [10-12]. Several investigators have now reported on the successful implementation of MC in clinical settings [10-25]. As a result, MC dose-calculation algorithms have been implemented for dosimetric verification of IMRT patient treatment plans.

One such work done by Yang et al.[14] investigated the accuracy of the CORVUS finite size pencil beam algorithm to the MC method for thirty prostate step-and-shoot IMRT plans utilizing both coplanar and non-coplanar beam arrangements. Their work, however, did not compare the MC re-calculated IMRT plans with measurements. MC calculations were preformed using EGS4/PRESTA code[13,20]. Their work compared the differences between CORVUS generated and MC recalculated IMRT plans in terms of differences in isodose distributions and dose volume histograms (DVHs). Their MC dose calculations, as compared to the CORVUS pencil beam algorithm, showed that while the differences in minimum target dose without heterogeneity corrections between two algorithms were within 4%, the differences in maximum dose to the bladder and rectum were <4% for twenty-five coplanar beam plans. For IMRT plans with non-coplanar beam arrangements without heterogeneity corrections, their results showed that the differences between MC and Corvus calculated doses to the CTV were >3% for all cases. For some cases, >9% differences in the minimum target dose was observed. The authors elaborated that this was probably due to the excessive attenuation of non-coplanar beams through the femurs. When the CORVUS heterogeneity corrections were turned on, the differences in mean target dose between MC and CORVUS were reduced to ~4%. The authors suggested that the IMRT plans utilizing non-coplanar beam arrangements should use heterogeneity corrections during treatment planning.

Another work done by Wang et al.[15] utilized MC calculation to evaluate the dosimetric effects of inhomogeneities for five clinical lung and five H&N IMRT plans. The IMRT plans were optimized using an in-house optimization algorithm utilizing an equivalent path length-based inhomogeneity correction and the plans were calculated using an in-house pencil beam dose calculation algorithm. All plans were recalculated with an EGS4-based MC calculation algorithm. Although most of the dose-volume indices calculated with both dose calculation algorithms agreed well, there were >5% differences for some plans.

Another work done by Sakthi *et al*.[16] evaluated the dynamic MLC IMRT dose-distributions calculated by the Pinnacle^3 ^system's (Philips medical Systems, Milpitas, CA) SC algorithm with EGS4-based MC calculations for twenty-four H&N patients treated with the SIB IMRT technique. Their work showed that the flat phantom measurements agreed much better with MC as compared to SC. They also observed that although average SC-computed doses in the patient agreed with MC-calculated doses, differences >5% between the two algorithms were common. They concluded that the inaccuracies in fluence prediction were the major source of discrepancy.

A work by Leal et al.[21] investigated the use of MC for routine IMRT verification. The IMRT plans were optimized using Plato TPS (Veenendall, the Netherlands) and the plans were recalculated using an EGS4-based MC system for three cases, including two prostate and cavum. The film dosimetry-based verification was also performed. Major differences were found between MC and TPS calculated doses in situations of high heterogeneity.

A study by Francescon et al.[22] compared the differences between step-and-shoot IMRT dose distributions calculated by the Pinnacle^3 ^system's (Philips medical Systems, Milpitas, California, USA) collapsed cone convolution algorithm (version 6.0i) with EGS4-based MC calculations for one prostate and one H&N case. The BEAM [17] MC code was utilized to simulate the particles through MLC. They found that the dose differences at the isocenter between Pinnacle^3 ^and MC calculations were 2.9% for H&N plan and 2.1% for prostate plan. However, there were up to 6% deviations for doses below 85% of the prescription dose and even much higher deviations for doses over the 85% of the prescription dose.

Another work done by Boudreau *et al.*[24] compared the dose distributions calculated with the CORVUS finite size pencil beam algorithm to the PEREGRINE MC dose calculations for eleven head and neck (H&N) patient treatment plans. Their MC dose calculations, as compared to the CORVUS pencil beam algorithm, showed that there was an average reduction of 16% and 12% in the GTV and CTV volumes covered by the prescription dose, respectively. They concluded that the differences between the CORVUS and PEREGRINE calculated doses were due to the lack of secondary electron fluence perturbations which are not modeled in the CORVUS, issues related to organ delineation near air cavities, and differences in reporting dose to water versus dose to medium.

The use of an algorithm such as MC, which can explicitly account for MLC leakage and scatter, can not only improve dose calculation accuracy, but also reduce the potential errors in the actually delivered dose to the patients. Although many successful implementation of MC in clinical settings have been previously reported [10-25] none of these work reported the MC verification of SIB-IMRT based prostate plans for a large set of patients. The SIB-IMRT generated treatment fields often have large intensity gradients which result in complex MLC leaf patterns and presents challenges to conventional dose calculation algorithms. The purpose of this study is to evaluate the dosimetric differences between Superposition/Convolution (SC) and Monte Carlo (MC) calculated dose distributions for twenty-two prostate patients treated with SIB IMRT dose distributions. Furthermore, the SC and MC calculated dose distributions were also compared to film-based measurements performed in phantom. The results of these comparisons will allow quantitative assessment of the dosimetric accuracy of prostate patients treated with SIB IMRT.

## Methods

### Patient Selection, Positioning and CT scanning

Twenty-two intermediate risk prostate cancer patients with the pelvic lymph node involvement that were treated with our in-house Internal Review Board-approved SIB IMRT protocol were selected for this study. Patients were CT scanned in a supine position with 3 mm slice thicknesses and slice separation using a Philips AcQsim scanner (Philips Medical Systems, Cleveland, Ohio, USA).

### Target volumes

The delineation of target(s) and critical structures for all patients was done by a single physician with extensive experience in the treatment of prostate cancer. For all patients, the clinical target volume (CTV) included 2 cm of seminal vesicles of the peri-prostatic rectum and a 5 mm expansion of the gross tumor volume (prostate only) in all directions, except posteriorly. The prostate planning target volume (PTV_prostate_) was generated expanding the prostate CTV by a uniform 5 mm in all directions. The nodal CTV included a 1 cm expansion of pelvic lymph nodes in all directions excluding the anterior portion of 1 cm skin, prostate PTV, bladder, rectum, small bowel, and bones. The nodal PTV volume (PTV_nodes_) was formed expanding the nodal CTV by 5 mm in all directions excluding prostate PTV and anterior skin 1 cm.

### Critical Structures

The critical structures included rectum, bladder, small bowel and femurs. Anterior portion of 1 cm skin region was also contoured and included in the optimization to limit dose to the anterior portion of patient's skin. In addition, the unspecified tissue was also contoured and included in the optimization.

### IMRT Optimization and Treatment Planning

All IMRT plans were generated using seven equally-spaced 18 MV coplanar beams for dynamic delivery with the Varian 21EX accelerator equipped with 120-leaf millennium MLC. The choice of the beam arrangements was based on the preliminary planning studies done for prostate IMRT patients. The prescription doses to PTV_prostate _and PTV_nodes _were 61–63 Gy and 50.4 Gy respectively, delivered simultaneously in 28 fractions, following an upfront 6 Gy high dose rate (HDR) brachytherapy. The Nominal Tumor Dose (NTD) at 1.8 per fraction was 76 Gy assuming a α/β = 3 for the prostate. The goal was to cover >97% of PTV_prostate _with 61–63 Gy and >95% of PTV_nodes _with 50.4 Gy. Dose-volume constraints for the critical structures were summarized in Table 1.

**Table 1:** Dose-volume constraints used in IMRT optimization and plan evaluations for twenty-two prostate patients.

***Structures***	***Limiting Dose(Gy)***	***Volume Constraint (%)***
**PTV**	61–65	97
	70	1
**PTV_Nodes_**	50.4	95
	60	5
**Femurs (L&R)**	35	50
	40	10
	45	2
**Rectum**	45	50
	60	10
	65	2
**Bladder**	45	50
	60	10
	65	2
**Small Bowel**	25	50
	45	10
	50	2
**Skin 1 cm Ant**	45	2
	30	20

Intensity modulation was achieved using the sliding window technique [26] which was implemented in the VCU in-house IMRT optimization system. For the SC dose calculation algorithm, the leaf positions (trajectories) are converted into energy fluence transmission maps by using an in-house analytic method that was based on the trajectory-to-fluence algorithm [27]. The energy fluence transmission maps were utilized to mainly attenuate the non-modulated open field energy fluence, thereby resulting in dose intensity modulation. The analytic algorithms often use simplifications in describing the MLC leaf geometry when determining the MLC transmission factor and leaf-end-modeling. This causes inaccurate representation of the fluence modulation produced by the MLC. The analytic trajectory-to-fluence algorithm utilized in this work included the average rounded leaf-tip transmission, which was determined from published MC simulation work, thus including head-scattered photons in the leaf-tip transmission and source size effects and also MC-derived term[11] that accounts for the scattered photons initiating from the MLC leaves. The in-house leaf sequencing method used for the SC algorithm in this work is also the basis of the dynamic MLC implementation in the Pinnacle^3 ^IMRT software module (7.4 and higher versions). The details of the leaf-sequencing method have been described in the literature[16,25,28].

During IMRT optimization, dose calculation was done using the SC algorithm available in Pinnacle^3^, with the intensity modulation determined as a transmission compensator matrix which was imported from the VCU IMRT optimization system. The optimized transmission compensator matrix, then, converted into a MLC leaf sequence as deliverable MLC transmission compensator matrix, which approximately accounts for the head-scatter, inter-leaf and intra-leaf leakage effects on the energy fluence. The deliverable fluence matrix, then, loaded into the Pinnacle TPS and the dose (caused by that energy fluence) within the patient was computed by the Pinnacle's SC algorithm.

The VCU in-house IMRT optimization system used in this study was interfaced with the Philips Pinnacle^3 ^TPS (Philips Laboratories, Milpitas, California, USA), that is used for contouring, beam placement, isodose display, and plan evaluation. The IMRT optimization system employed a gradient-based search algorithm and described in detail elsewhere [29]. The Pinnacle's adaptive SC dose calculation algorithm, including heterogeneity corrections, which was based on the work done by Mackie et al.[4,30], was used during both optimization and final dose calculation stage after MLC leaf sequencing was performed. Our numerical experiments did not find any difference between Pinnacle's collapsed-cone and adaptive SC results and therefore, adaptive SC was used for treatment planning of all clinical patients. The adaptive SC dose calculation algorithm model consists of 1) modeling the incident energy fluence as it exits the accelerator head, 2) projection of this incidence energy fluence through a density representation of a patient to compute a total energy released per unit mass (TERMA), and 3) 3-D superposition of the TERMA with an energy deposition kernel to compute the dose. The algorithm also uses a ray-tracing during superposition to incorporate the effects of the heterogeneities to the lateral scatter. The Pinnacle's adaptive SC beam model parameters characterize the radiation exiting the head of the linear accelerator by the starting point of a uniform plane of energy fluence describing the intensity of the radiation. The algorithm, then, adjusts the fluence model to account for the flattening filter, collimators and beam modifiers. The SC beam modeling requires the measurements of the depth dose curves (the energy spectrum determination), dose profiles (incident energy fluence determination inside the field), dose profiles extending outside the field (scatter dose determination from the machine head components), calibration and relative output factors. The initial energy spectrum for 6 MV and 18 MV photon beams was chosen from a library of spectrums available in Pinnacle^3 ^beam modeling module. The dose calculation grid for each IMRT patient plan included the entire patient CT data set and was 4 mm in each Cartesian coordinates. The adaptive SC algorithm was commissioned to match measurements, and the agreement between the measurements and the adaptive SC were generally within ± 2% or 2 mm for both open and MLC-defined fields. The Pinnacle^3 ^beam modeling measurements were performed in a Wellhofer 48 cm × 48 cm × 48 cm water phantom (IBA Dosimetry, Bartlett, Tennessee, USA) for field sizes ranging from 1 cm × 1 cm to 40 cm × 40 cm. The measurements of 5 cm × 5 cm to 40 cm × 40 cm field sizes were performed using Wellhofer IC-10 (0.1 cm^3 ^active volume). For the measurements of small field sizes of 1 cm × 1 cm to 4 cm × 4 cm, Wellhofer IC-3 chamber (0.03 cm^3 ^active volume) were used.

### Monte Carlo Dose Verification

SIB IMRT plans for each patient in this study were recomputed with MC to investigate the accuracy of the SC algorithm which was coupled with our in-house SC fluence modulation prediction algorithm. MC dose recalculation for each patient was performed using the same leaf sequence files and monitor units (MUs) that were obtained using SC based optimization. Hence, the MC results were computed in terms of dose per MU and the MUs used for the patient's treatment were the ones used for the dose evaluation. The SC method (as described in IMRT optimization and Treatment Planning section) converts the MLC leaf sequencing file into a virtual compensator to perform the IMRT calculations, whereas the MC method uses the MLC leaf sequencing file directly. The strength of MC-based methods stems from the fact that it can realistically model radiation transport and interaction process through the accelerator head, beam modifiers and the patient geometry [10]. Specifically, the MC calculation algorithms can include the detailed description of the MLC leaf geometry and directly consider the effect of the MLC on the primary and scatter beam fluence on a particle-by-particle basis. The implementation of the MC algorithm used in this work was described in detail elsewhere[16,25], but is briefly summarized here for completeness. Our MC dose calculations were based on EGS4 code [31], along with user codes BEAM [17] and DOSXYZ [32]. The accuracy of EGS4 code, along with user codes BEAM and DOSXYZ, for both homogeneous and heterogeneous phantoms have been extensively tested by other investigators [12,19,33] hence will not be discussed here. The MC simulations were run on a dedicated dual-processor Beowulf cluster, containing ten 2.4- to 2.8-GHz dual-processor nodes. MC algorithm is interfaced to Pinnacle^3 ^TPS such that an integrated control interface directly reads gantry angles, jaw positions, beam energies, and patient CT densities from the Pinnacle^3 ^TPS. Particles in each beam during MC simulation were read from a previously-commissioned phase-space that includes particle positions, directions, and energies exiting the treatment head which are incident upon the MLC using BEAM [17], through the dynamic MLC using an in-house code [10], and through the patient using DOSXYZ [32], where deposited energy was scored. In the MC MLC model, the MLC was divided into simple geometric regions where the simplified radiation transport can be performed. For photon beams, the MC MLC model predicted both beam hardening and leaf-edge effects (tongue-and-groove) and included attenuation and first Compton scatter interactions. The MLC leaf positions were directly read from the MLC leaf sequence files that are generated by the IMRT optimization system. The positions in the leaf sequence files were then translated into physical MLC leaf tip positions at the MLC plane using a look-up-table and demagnification from the machine mlctable.txt file. After the MLC leaf tip positions, as a function of monitor units, are determined, the particles were transported from the phase-space of particles leaving the treatment machine jaws and the particles were transported through the MLC leaves. The particles exiting the MLC were written into a phase-space file which was used as an input for MC patient dose calculation. The MC MLC method summarized here was tested for both 6-MV and 18-MV photon beams and the details of this method have been reported in the original paper by Siebers et al. [10].

For MC calculations, the dose calculation grid for each patient included the entire patient CT data set and was 4 mm in each x, y, and z Cartesian coordinates. For each beam, a nominal value of ~2% statistical uncertainty at a depth of D_max _was used for all MC dose calculations, leading to a 1% overall statistical uncertainty from all treatment beams in the dose to the target structures. It has been previously shown that an overall 2% statistical uncertainty in MC calculations has minimal effect on DVHs [12,34]. Structure-by-structure analysis of the statistical uncertainty in the dose to the critical structures was <1.5% respectively [35] for the prostate cases included in this study. The uncertainty in DVH-evaluated parameters, however, was <1.0%. Pinnacle SC dose calculation algorithm utilized in this work reports absorbed dose to water. The MC dose calculation algorithm, on the other hand, inherently reports absorbed dose to medium. For consistency with SC calculations, the MC calculated dose distributions were converted from dose-to-medium to dose-to-water using the post MC-calculation methods described in Siebers *et al.*[36]

The MC dose calculation algorithm used in this work has been commissioned to match measurements and has been thoroughly tested and benchmarked against measurements for both 6-MV and 18-MV photon beams. The details of the MC commissioning can be found in the references [10-12,37-39]. The agreement between our MC dose calculation with the measurements for both open and dynamic MLC-defined fields was found to be generally within ± 1% or 1 mm [10,12], except in the build-up region and for very small sliding window DMLC fields (0.5 cm) where there were disagreements up to 1.5% (for 6 MV) and 2.5% (18 MV) between MC calculated and the measured doses.

### Comparison with Film Measurements

In addition, the patient plans that were initially planned and treated using VCU IMRT system were experimentally verified beam-by-beam using film dosimetry as part of the routine IMRT QA. The verification of each dose calculation algorithm for each treatment beam (verification of the IMRT fluence estimation) was quantified by performing dose calculations using both SC and MC algorithms in a flat water phantom. The SC and MC calculated dose distributions results were compared to EDR2 film measurements (Eastman Kodak, Rochester, New York, USA) performed at a 5 cm depth in a 30 cm × 30 cm × 20 cm solid water phantom. For EDR2 film measurements for each plan, the gantry angles were set to zero (Varian system) with a source to film distance 100 cm. The film measurements utilized the same MLC leaf sequence files that were used for the patient IMRT treatment as well as used in SC and MC recalculation.

For each patient treatment plan, the film calibration curves were generated by irradiating films, placed at *d*_max_, 100 cm SSD, 10 × 10 cm field (where 1 MU = 1 cGy), with 0 to 300 MUs in increment of 10 MUs. EDR2 films used for the measurements of treatment and calibration films came from the same batch. All films for each plan were processed the day of irradiation and scanned using the VIDAR VXR 16 (Vidar Systems Corporation, Herndon, Virginia, USA) and were analyzed with an in-house developed scanning software. The reproducibility of the films was within 0.5%. The measured dose distributions of each beam were superimposed with the SC and MC calculated dose distributions and the parameters such as gamma index, dose difference, distance-to-agreement (DTA) were calculated using an in-house software developed based on the published work by Low et al.[40] and Harms et al.[41] For each plan, both the 2% dose difference, 2 mm DTA criteria and the 3% dose difference, 3 mm DTA criteria were used for the calculation of the fraction of points passing with gamma (γ) index <1.

### Plan evaluation

For each patient plan, the SC and MC calculated patient plans were evaluated using dose-volume-based indices (see Table 1). For the PTV_prostate_, the minimum dose received by 98% of the volume (D_98_), the maximum dose received by 2% of the volume (D_2_), the dose received by 50% of the volume (D_50_) and the mean dose (D_mean_) were evaluated. For PTV_nodes_, the minimum dose received by 95% of the volume (D_95_), D_50 _and D_2 _were evaluated. For critical structures, D_2_, D_10_, D_50 _indices were evaluated. The D_2 _index was used as a surrogate to evaluate the maximum dose since in some plans, volumes of only a very small number of voxels received higher or lower doses and this overstated the absolute maximum and minimum dose, and could bias the data. Furthermore, the D_2 _is less prone to the statistical fluctuations in MC methods [16].

The dose-volume constraints for all structures defined in Table 2 were used for the evaluation of all plans. In addition, the homogeneity index (HI), which was defined as the [(D_98 _- D_2_)/D_presc_.], was calculated for PTV_prostate_. The comparisons between SC optimized and MC re-calculated plans were made relative to the SC calculated plans using a paired two-tailed student's t-test. The average values of the dose-volume indices were found to be statistically significant if p value ≤ 0.05. For each patient, differences between the SC and MC re-calculated plans were calculated with respect to the local point of interest using the formula:

**Table 2:** Summary of results for twenty-two prostate plans, showing average relative % differences between SC and MC calculated dose-volume indices including standard deviation and the range of dose-volume indices.

**Structure**	**Dose-volume index**	**Range of Indices (%)**	**Average relative % difference**
**PTV_prostate_**	D_98_	[-3.8, 0.06]	1.2 ± 1.1 (p < 0.05)
	D_50_	[-3.2, 0.9]	0.8 ± 1.0 (p < 0.05)
	D_2_	[-0.5, 4.2]	1.4 ± 1.4 (p < 0.05)
	D_mean_	[-2.5, 2.9]	0.3 ± 1.1 (p > 0.05)
**PTV_nodes_**	D_95_	[-4.7, 0.6]	1.5 ± 1.4 (p < 0.05)
	D_50_	[-3.0, 1.0]	0.4 ± 1.3 (p > 0.05)
	D_2_	[-2.1, 2.6]	0.1 ± 1.1 (p > 0.05)
	D_mean_	[-2.9, 1.4]	0.3 ± 1.2 (p > 0.05)
**Rectum**	D_50_	[-3.6, 3.4]	0.2 ± 1.8 (p > 0.05)
	D_10_	[-2.9, 2.2]	0.3 ± 1.5 (p > 0.05)
	D_2_	[-2.5, 1.9]	0.4 ± 1.2 (p > 0.05)
	D_mean_	[-2.9, 3.1]	0.6 ± 1.7 (p > 0.05)
**Bladder**	D_50_	[-3.7, 1.4]	0.9 ± 1.4 (p < 0.05)
	D_10_	[-3.2, 1.5]	0.7 ± 1.3 (p < 0.05)
	D_2_	[-2.7, 2.2]	0.7 ± 1.1 (p < 0.05)
	D_mean_	[-3.8, 0.9]	0.8 ± 1.3 (p < 0.05)
**Small Bowel**	D_50_	[0.2, 115]	30.2 ± 40.7 (p < 0.05)
	D_10_	[-3.1, 120.7]	10.1 ± 26.4 (p < 0.05)
	D_2_	[-2.6, 100.1]	6.8 ± 21.5 (p < 0.05)
	D_mean_	[-1.5, 123.5]	16.5 ± 27.5 (p < 0.05)
**Femurs**	D_50_	[1.2, 14.5]	8.6 ± 3.6 (p < 0.05)
	D_10_	[-3.0, 8.4]	4.6 ± 3.5 (p < 0.05)
	D_2_	[-2.9, 8.6]	4.1 ± 3.3 (p < 0.05)
	D_mean_	[-0.9, 10.3]	6.3 ± 3.9 (p < 0.05)
**Skin 1 cm Ant**	D_2_	[1.7, 12.1]	7.7 ± 3.8 (p < 0.05)

The p values determine if the mean of relative differences are significantly different from zero.



where x is a particular dose-volume index and SC and MC are the techniques being evaluated. The comparisons were made relative to the SC calculated plans since these plans were used for the patient treatments.

In addition to dose-volume indices, the SC- and MC-calculated 3D dose distributions were compared using the 3D gamma analysis [42] with the gamma criteria of 3% dose difference and 3 mm DTA. The MC dose calculation was used as the reference dose for the 3D gamma analysis. For both SC and MC dose calculations, the dose calculation grid size was set to 0.4 cm × 0.4 cm × 0.4 cm. For 3-D gamma index calculation, the dose values were interpolated linearly at a sample step size of 0.02 cm. The maximum search distance was set to 1.0 cm. When a sample step size of 0.02 cm was used during the linear interpolation, the differences in the percentage of the points passed the gamma criteria was very negligible for the dose calculation grid sizes of 0.4 cm, 0.3 cm and 0.2 cm. This is also consistent with the results presented at the work done by Wendling et al. [42]. For each structure, the gamma values averaged over all patient population were computed.

## Results

### Monte Carlo Verification of Film Measurements

**Figure 1:**
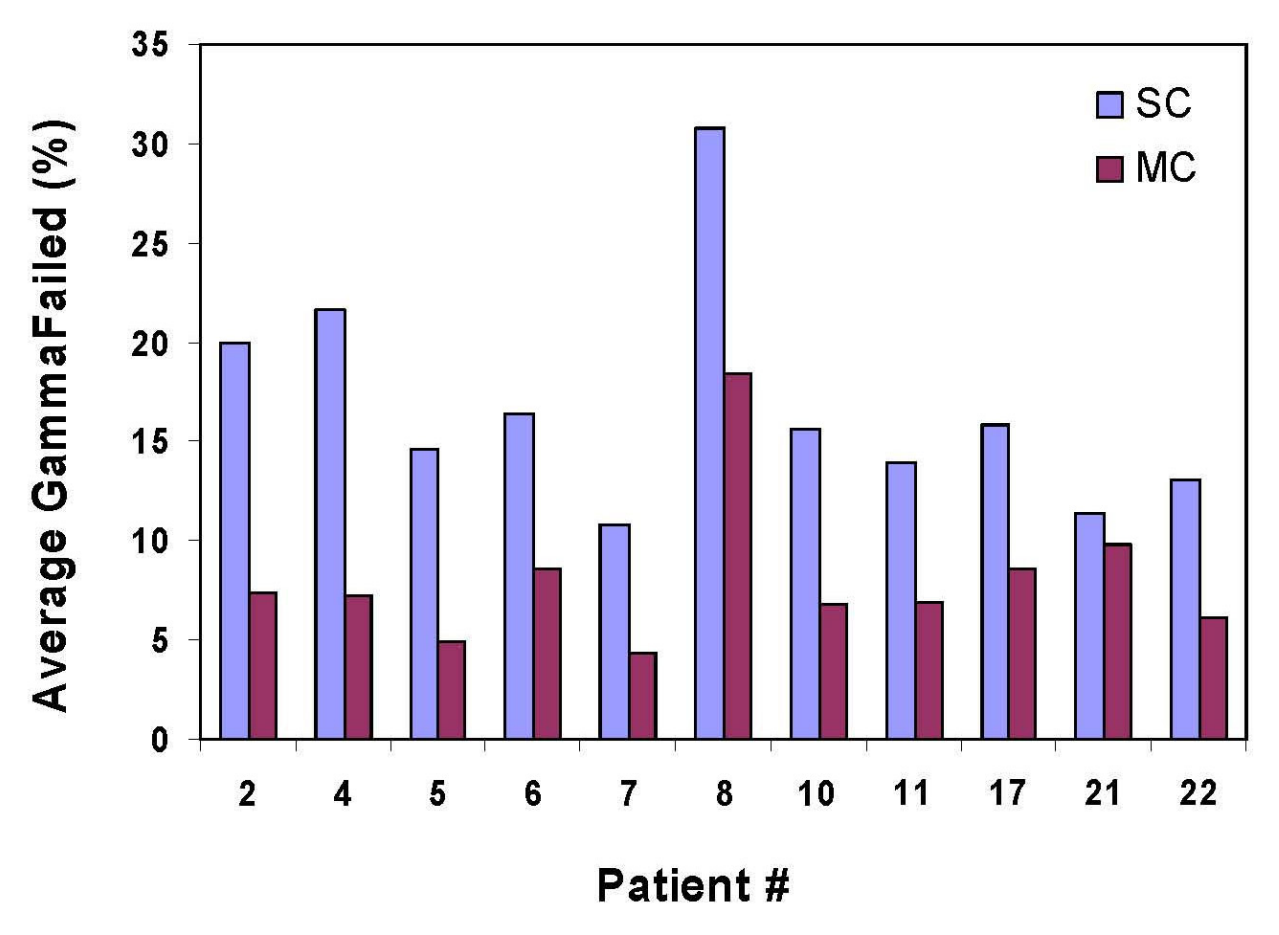
**Gamma analysis that compares SC and MC algorithms with measured dose distributions in flat phantom for 11 of patient plans**. The percentage of points failed was averaged over all of the fields for each patient for γ >1 with 2% tolerance and 2 mm DTA. The agreement of MC results is better than SC.

Figure 1 summarizes the gamma analysis of eleven of the patient plans (included the ones with the highest and lowest percentage of points failed gamma criteria) by comparing the phantom measured dose distributions with SC and MC calculated dose distributions. In Figure 1, the percentage of points failed gamma test were performed averaged over all of the plan's treatment fields for each patient with γ >1 with 2% tolerance and 2 mm distance to agreement (DTA). The results demonstrate that the average of patient plans with percentage of points failing gamma test is 8.1% ± 3.8% for MC (ranging from 4.3% to 18.4%) and 16.7% ± 5.7% for SC (ranging from 10.9% – 30.7%). For a more commonly used clinical gamma criteria of 3%/3 mm, the average of patient plans with percentage of points failing gamma (γ >1) was 2.6% ± 1.6% for MC (ranging from 1.3% to 5.7%) and 5.2% ± 3.8% for SC (ranging from 2.0% – 12.6%).

**Figure 2:**
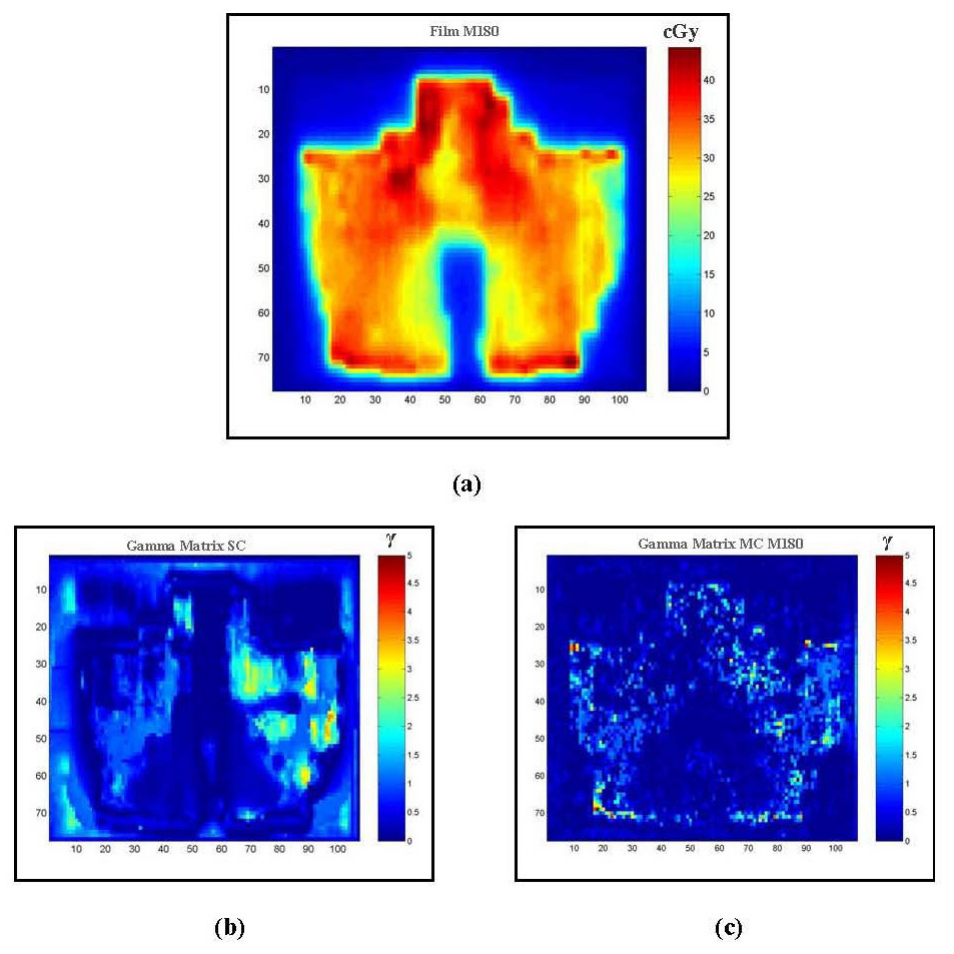
**(a) The measured dose distribution and gamma analysis that compares with the (b) SC and (c) MC calculated dose distributions in flat phantom for one of the patient treatment fields**. The percentage of points passed was calculated for γ <1 with 2% tolerance and 2 mm DTA. The agreement of MC results is better than SC. Note that the color bar in (a) represents the measured dose in cGy, whereas the color bar in (b) and (c) shows the range of γ values.

Figure 2a–c shows gamma analysis comparing the measured dose distribution with the SC and MC calculated dose distributions in flat phantom for one of the patient treatment fields (180° angle). The percentage of points passed for γ <1 with 2% tolerance and 2 mm DTA was 91.2% with MC (Figure 2c) as compared to 84.3% for SC (Figure 2b).

### Monte Carlo Verification of Patient Plans

**Figure 3:**
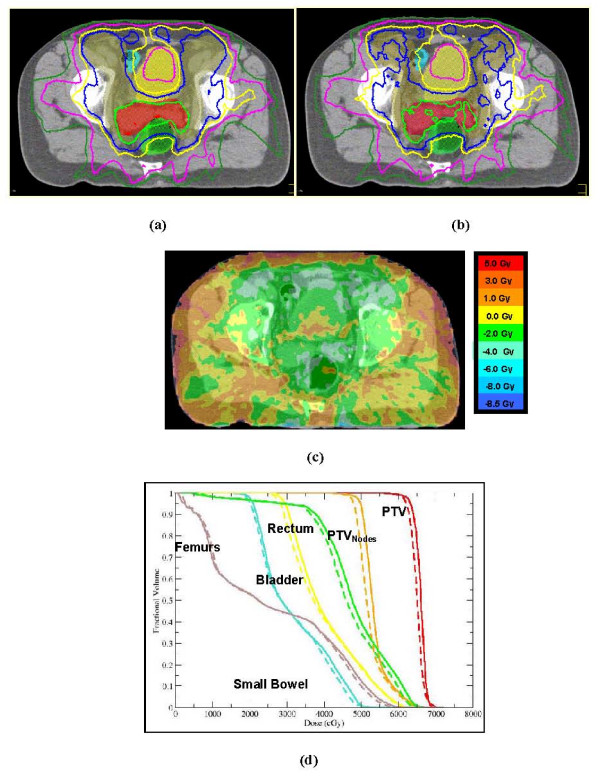
**Comparison of (a) SC- and (b) MC-calculated isodoses on transverse CT slice, (c) colorwash of absolute dose differences between two methods and d) DVHs for Patient 12 included in this study**.

Figure 3a–d shows the comparison of SC and MC calculated transverse-slice isodose distributions and the corresponding absolute dose differences between the two dose-calculation algorithms. Also shown, are the DVHs for one of the patients (Patient 12) included in this study.

While the approved plan (SC calculated) for this patient delivered 62 Gy to 98% of the PTV_prostate_, the MC re-computed PTV D_98 _predicted 61.2 Gy (1.39% lower than the predicted by the SC). The MC predicted PTV_prostate _D_50 _was also 1.6% lower than the one predicted by SC. For PTV_nodes_, the MC predicted D_95 _(47.9 Gy) was 3.5% lower than the one predicted by the SC (49.7 Gy). The value of this index was slightly higher than our clinically acceptable tolerance level of 3%. The HI for PTV_prostate _increased from 10.1 with SC to 11.6 with MC for this patient. For critical structures, the MC also predicted lower doses for each dose-volume index. These differences were <3.5%. With the exception of the PTV_nodes_, the differences between SC and MC predicted dose-volume indices for all PTVs and critical structures were in general within our clinically acceptable tolerance level of 3%. Figure 3c displays the absolute dose difference between the SC and MC calculation algorithms on a transverse plane for Patient 12. The range of dose differences between the two calculation methods varied from -8.9 Gy to +5.0 Gy, showing greater positive deviations in regions close to the patient skin, and in regions where heterogeneity structures (e.g., bone, air) and also where large intensity gradients present. The greater positive deviations in skin were due to less accurate prediction of surface doses and doses in build-up region by SC algorithm as compared to MC algorithm. Figure 3d displays the DVHs calculated with SC (solid lines) and MC (dashed lines) for this patient, showing lower MC doses for all structures.

Dosimetric results for all patients are summarized in Table 2, which shows the average relative % differences with their standard deviations and ranges for the dose-volume indices for twenty-two IMRT patient plans. On average, MC predicted doses for PTV_prostate _and PTV_nodes _were lower than the ones predicted by SC, indicating ~1.6% systematic difference in the SC calculated dose. Although the average relative % difference between SC and MC calculated D_98_, D_50_, D_2 _and D_mean _indices for PTV_prostate _is less than 1.5%, there were deviations up to 4.2% in the regions of prostate PTV extending to the bone, in individual patient plans (Patient 15). Similarly, although the average differences in SC and MC calculated dose-volume indices for PTV_nodes _were less than 2%, deviations as high as -4.7% (Patient 11) in areas where the PTV_nodes _volume extending to the anterior portions of the skin region. For both rectum and bladder, the average relative % differences for all dose-volume indices were less than 1%; however, differences up to 3.8% in bladder D_mean _were observed in some patients (Patient 8). The reason for this large difference in bladder D_mean _may be due to the large air pocket in the bladder of this patient which was introduced by pulling of the foley catheter before the CT scanning. The largest differences between SC and MC computed doses were observed in small bowel and femur. Differences of 0.2% to 115% in small bowel D_50 _(e.g.; Patient 14) and 1.2% to 14.5% in femurs D_50 _were observed. The large deviations in small bowel doses was due to large differences in small bowel volume within the treatment field and very small doses received by the small bowel for some patients. Since the small bowel volume for Patient 14 was very small (14 cc) and was far from the high dose regions, it received much lower doses as compared to the other patients (e.g; D_50 _= 104.7 cGy with SC vs. 225.1 cGy with MC). Therefore, the observed large differences are as a result of the large MC statistical uncertainties in this lower dose region.

For majority of patients, large differences in SC and MC calculated dose-volume indices for femurs may be due to the systematic errors introduced when converting from dose-to-medium to dose-to-water in MC-calculated IMRT treatment plans. For a previously done study on these prostate IMRT patients [35], for femoral heads, the systematic shifts of ranging from 4.0% to 8.0% in dose-volume indices were observed when using dose-to-water vs. dose-to-material. This systematic shift for femurs was due to the high calcium content of the bones, which increased the water-to-material stopping power ratio due to the increased neutron/proton ratio in calcium relative to water (caused by the hydrogen content of water).

**Figure 4:**
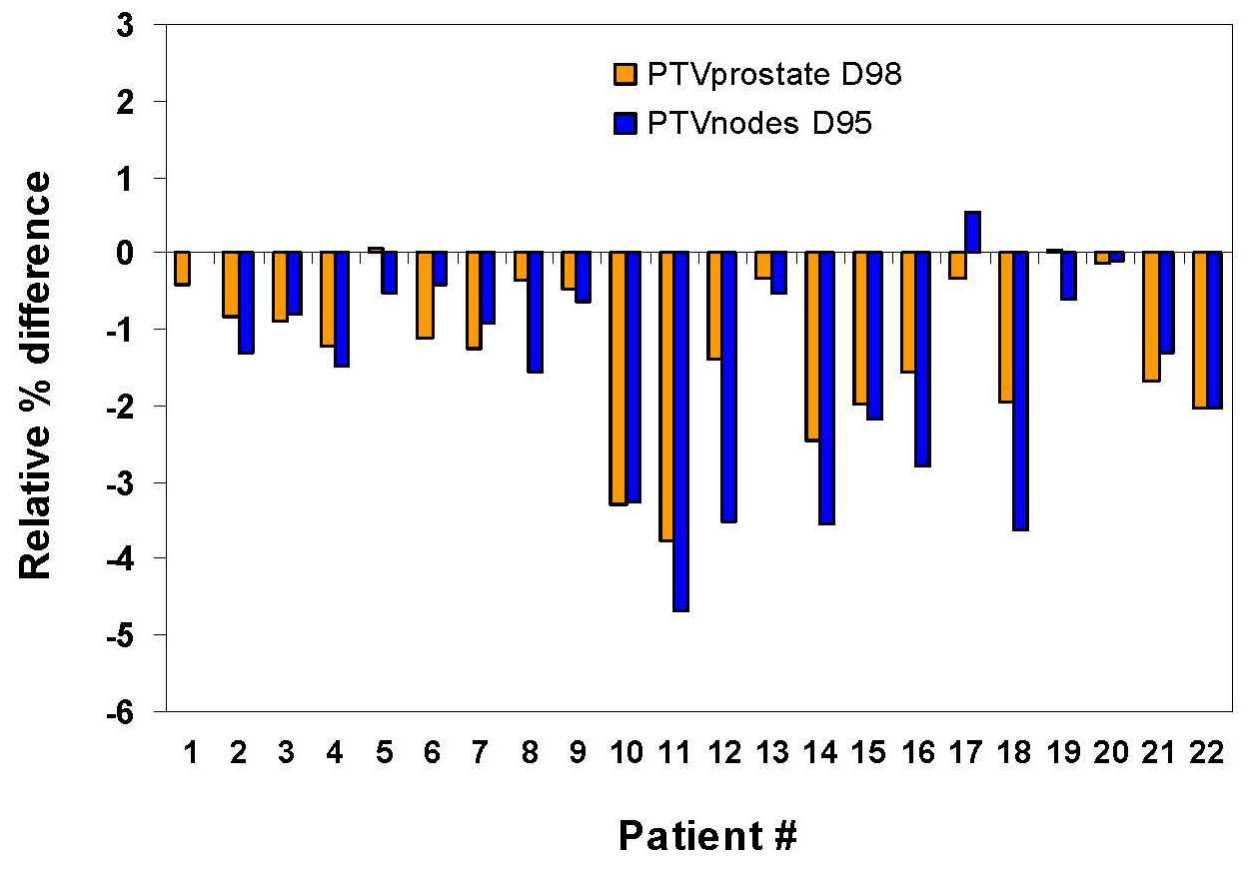
**Relative percent differences between SC and MC calculated plans for the PTV_prostate _D_98 _and PTV_nodes _D_95_**. MC predicted doses are lower than SC doses for all, except for two patients. Note that relative % difference =  where x is a specified dose-volume index.

Figure 4 graphically illustrates the patient-to-patient variation in relative percent deviations between SC and MC computed PTV_prostate _D_98 _and PTV_nodes _D_95 _indices. The deviations in PTV_nodes _D_95 _were greater than PTV_prostate _D_98 _and the MC calculated doses were lower than the ones initially predicted by SC, except for two patients.

**Figure 5:**
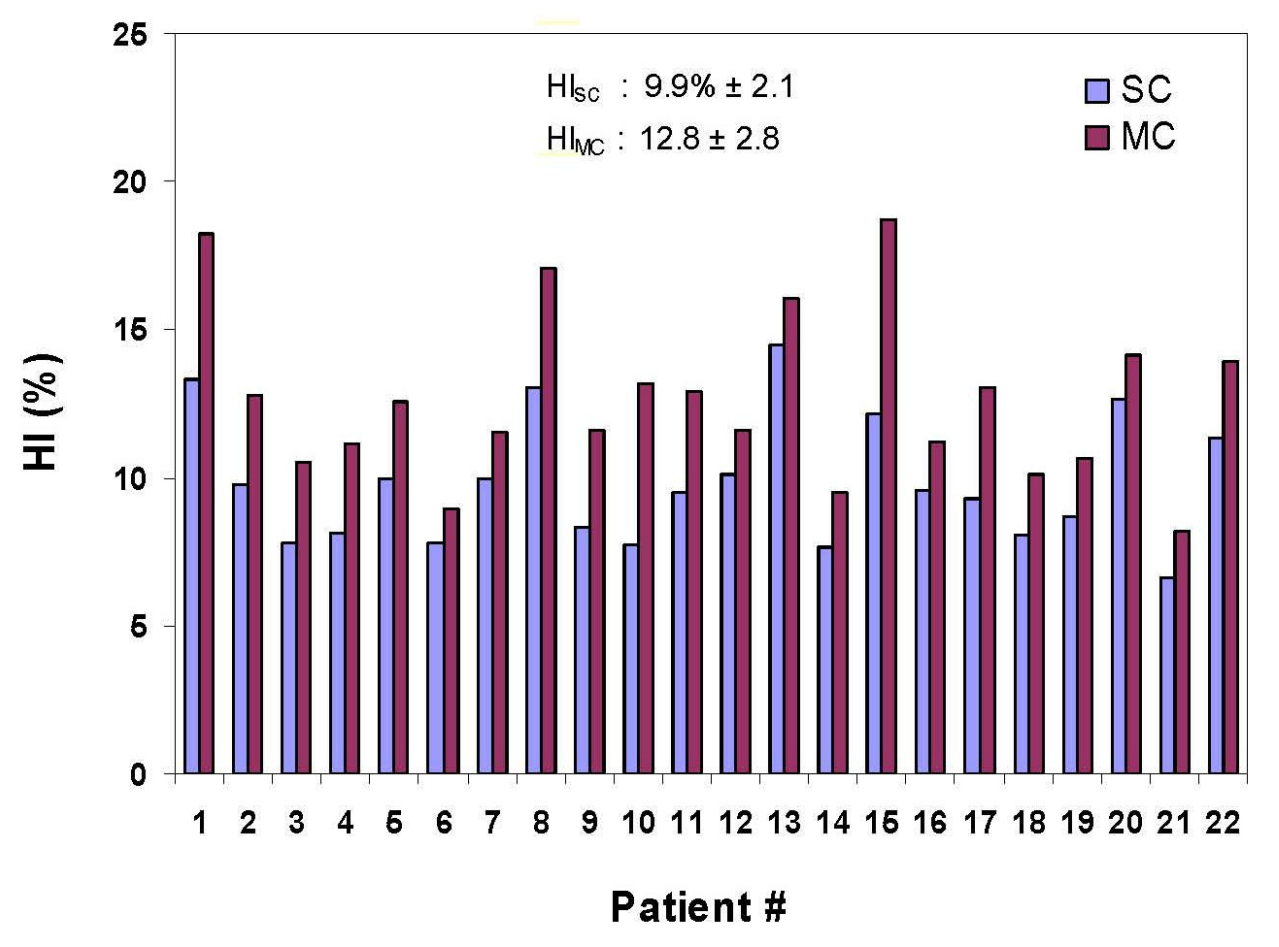
**Prostate PTV homogeneity indices (HI) with SC and MC computed IMRT plans for all patients**.

Figure 5 shows the patient-to-patient variation in SC and MC calculated homogeneity index (HI). For all plans, MC recalculated plans were less homogeneous than the planned SC dose distributions (average HI: 9.9% ± 2.1 for SC vs. 12.8 ± 2.8 for MC). The largest difference in HI between the SC and MC calculated plans was found to be for Patient 10 (13.2 for MC vs. 7.7 for SC).

**Figure 6:**
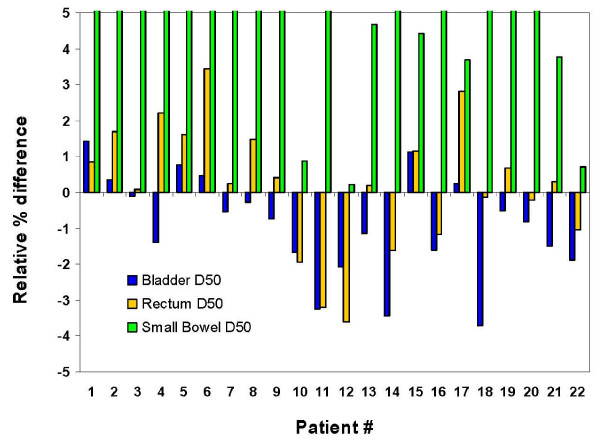
**Relative percent differences between SC and MC calculated plans for the bladder, rectum and small bowel D_50_**. Relative percent differences between SC and MC calculated plans for the bladder, rectum and small bowel D_50_. Note that relative % difference = .

Figure 6 shows the percent deviations between SC and MC computed bladder, rectum and small bowel D_50 _for all patients. Although the deviations for both rectum and bladder were on both sides of the norm, the MC predicted doses were lower for the majority of the patients (15 out of 22) for the bladder and MC doses were higher for the majority of the patients (14 out of 22) for the rectum. The deviations between SC and MC computed dose-volume indices for bladder and rectum were within 3% for the majority of the patients. This is within our clinical acceptance criterion for our clinical IMRT patient dose verification protocol (≤ 3%). However, up to 6.5% differences were observed for some patients. For all patients, the percent deviations in small bowel D_50 _were positive, indicating that MC predicted doses were higher. However, the dose-volume criteria were still clinically acceptable by the physician since D_50 _values were within or well below the tolerance doses.

The evaluation of the number of patient plans that satisfied a given dose-volume criteria is presented in Table 3. For majority of the plans, the SC and MC calculated dose-volume indices agreed within 3% for both target and critical structures. For example, twenty out of twenty-two patients had the SC and MC calculated indices agree within 3% for both PTV_prostate _D_98 _and eighteen out of twenty-two plans agreed within 3% for PTV_nodes _D_95_. If we considered all of the dose-volume indices for all target structures (D_98_, D_95_, D_90_, D_50 _and D_2_), sixteen out of twenty-two patients were within 3% criteria. The rest of the plans were within 5% criteria. Although the similar agreement was observed for rectum, bladder and small bowel, five out of twenty-two plans exceeded the D_50 _= 25 Gy criteria for small bowel. However, the physician still considered these plans clinically acceptable.

**Table 3:** Number of patient plans satisfying a given dose-volume criteria for target and critical structures.

**Indices**	**Criteria**	**Number of Plans** Relative % difference
**PTV**_**prostate **_**D**_**98**_	<3%	20
	>3%, <5%	2
**PTV**_**prostate **_**D**_**50**_	<3%	21
	>3%, <5%	1
**PTVN**_**nodes **_**D**_**95**_	<3%	18
	>3%, <5%	4
**PTVN**_**nodes **_**D**_**50**_	>3%	21
	>3%, <5%	1
**Bladder D**_**50**_	<3%	19
	>3%, <5%	3
**Bladder D**_**2**_	<3%	22
**Rectum D**_**50**_	<3%	19
	>3%, <5%	3
**Rectum D**_**2**_	<3%	22
**Small Bowel D**_**50**_	<10%	5
**exceeding 25 Gy due to deviation**	>10%	1
**Small Bowel D_2_**	<3%	17
	>5%, <10%	1
	>10%	4

Table 4 summarizes of the average percentage of points passing the 3D gamma evaluation for the PTV, PTV_prostate_, rectum, bladder, small bowel and femurs and the total 3D dose distribution. While for PTV_prostate_, PTV_nodes_, bladder and rectum, the average gamma scores were >95.0%, they were 90.2% for small bowel and 91.6% for femurs. The lower 3D gamma pass rates for small bowel and femurs are consistent with the large differences observed in dose-volume indices between MC- and SC-calculated doses.

**Table 4:** Gamma index values averaged over all patient population for each structure and 3D total dose with gamma criteria of 3% dose difference and 3 mm DTA.

	**PTV_prostate_**	**PTV_nodes_**	**Rectum**	**Bladder**	**Small Bowel**	**Femurs**	**Total 3D dose**
**Fraction of points pass γ (%)**	96.0 ± 4.4	96.1 ± 3.9	97.7 ± 2.7	96.5 ± 4.8	90.2 ± 9.7	91.6 ± 5.7	94.3 ± 1.9

Note that The MC dose calculation was used as the reference dose for the 3D gamma analysis.

## Discussion

This study of twenty-two prostate patient cohort treated with SIB IMRT showed differences between the SC and MC predicted doses. Film dosimetry results confirmed that the MC algorithm predicted flat-phantom measurements better than the SC algorithm (Figure 1). Improved MC calculated dose agreement was due to the superior fluence prediction by the MC algorithm. For each patient plan, the deviations of the phantom and patient data sets for each case were found to be on the same side of the mean, demonstrating that the deviations observed in flat-phantom are representative of the deviations in the patients.

For individual patient plans, our results showed that the MC-predicted doses for PTV_prostate _and PTV_nodes _were up to 3.8% and 4.7% respectively lower than the ones predicted by the SC algorithm. For some patients, the magnitude of such deviations might decrease the intended target dose levels that are required for the treatment protocol, placing the patients in different dose levels that do not satisfy the protocol dose requirements. For rectum and bladder, the differences in SC and MC predicted doses were less than 3% for the most of the patient plans although deviations as large as 3.8% were seen some individuals. For femoral heads, the deviations between SC and MC predicted doses reached 14.5%. This appeared to be related to the calcium content of the bones.

In this work, differences between SC and MC predicted mean doses (D_mean_) for the PTV_prostate _were similar (<3.0% with an average of 0.3) to those reported by Yang et al.[14], whose work showed that the differences in mean dose to the prostate CTV were within 3%. In our study, differences in maximum dose (D_2 _was used as a surrogate) to the rectum and the bladder for all cases were ≤ 2.7% (with an average of 0.38% and 0.0.72% respectively). In contrast, the differences in maximum dose to the rectum in study by Yang *et al*.[14] were ≤ 4%. Other differences exist between these two studies. Yang *et al.*[14] used a step and shoot technique whilst the dynamic MLC (sliding window) technique was used in this work. Lastly, their work focused on the investigation of the effects of heterogeneities on dose distributions estimated by both pencil beam and MC algorithms using both coplanar and non-coplanar beams. Any of these factors could account for the differences observed in our study. Our work supports the fact that the dose prediction errors vary significantly (>3%) from patient to patient, suggesting that individual patient evaluation is indicated.

In this work, possible causes of differences between the SC and MC predicted doses were likely due to the inaccurate prediction of the beam fluences, imprecise handling of heterogeneities in patient, and differences in beam modeling for SC and MC dose calculation algorithms. A work by Mihaylov *et al.*[28] found that the differences in the DMLC modeling between MC-based methods and the commercial algorithms were main causes of dosimetric differences. Hence, we speculate that the main contributing factor to the dosimetric differences observed in this study is in the MLC transport rather than transport in the patient. In this work, for the highly modulated SIB IMRT prostate cases involving two targets (PTV_prostate _and PTV_nodes_) and critical structures, and our in-house analytic fluence prediction algorithm together with the Pinnacle's adaptive SC overestimated the MLC transmission, scatter and leakage, resulting in larger doses to both PTV_prostate _and PTV_nodes _as compared the doses calculated by MC algorithm, resulting in negative dose prediction errors.

The MC algorithm used in this work inherently included the leaf scatter, tongue-and-groove, and beam hardening effects on the fluence upon the patient. In the SC dose-calculation algorithm, however, the intensity modulation was incorporated into dose computation using a transmission-compensator matrix during the estimation of fluence upon the patient. The MLC leaf scatter, tongue-and-groove and beam hardening effects were approximately incorporated into the fluence modulation. The fluence-to-trajectory estimation that is more accurate than the one used for this work would improve the results of SC calculation of prostate SIB IMRT patients. The dynamic MLC leaf-sequencing technique utilized in this work is the basis of the sliding window technique used in Pinnacle^3 ^IMRT software (7.4 and higher versions) that includes the tongue-and-groove effect. Mihaylo *et al.*[28] compared the in-house fluence estimation used in this work with the one used in Pinnacle^3 ^dynamic MLC IMRT module, and his gamma analysis with ≤ 2%/2 mm criteria revealed that the results of both methods agreed within 1%.

The SC and MC algorithms used in this work utilized different fluence prediction models although both SC and MC algorithms[10,37] have been extensively tested and commissioned to match the measurements. Therefore, the use of these different beam models might explain the dose differences between SC and MC calculations. The contribution of use of different fluence prediction algorithms to the dose prediction errors is not included in this work and is the subject of a future study. One possible solution to avoid the differences introduced due to different fluence prediction models is to use the MC to predict the energy fluence and to use the SC to predict the dose to the patient. A work done by Mihaylov et al. [28] introduced a hybrid method, which utilized a MC code, to predict the energy fluence modulation incident upon a patient, and a conventional dose calculation algorithm (SC) to estimate the resultant dose within the patient. All computational methods used in their work use the same dose calculation algorithm to predict the dose to the patient, thereby isolating the effect of the prediction of the incident energy fluence which is inherent to the dose calculation algorithm used. They benchmark their method by comparing in-phantom measured dose distributions with analytic methods (the one used in our work), including the one implemented in Pinnacle^3 ^TPS. Their work showed that the hybrid method better predicts the measurements and also shows that the major factor causing the differences between the SC and MC was the estimation of the fluence upon the patient.

In Figure 3a–b and 3d, the effect of heterogeneities for Patient 12 is not very apparent. There was 3.4% decrease in maximum dose predicted by MC to the femoral heads for this patient, for some patients, however, there were up to 8.6% increase in D_2 _predicted by MC to the femoral heads, indicating that the heterogeneous patient medium can be a potential source. Dose deviations due the fractional contribution of patient heterogeneities for prostate SIB IMRT patients are the subject of a future study. A work by Mihaylov et al. [43] quantified the contribution of patient heterogeneities for head and neck SIB IMRT patients. Their work demonstrated that the effect of SC-modeled tissue heterogeneities were < ± 3% for 98.3% of the dose-volume indices used for the evaluation.

Differences between SC- and MC-calculated prostate SIB IMRT dose distributions indicates that the SC calculated treatment plans contain optimization convergence errors[25]. Clinically, the optimization convergence errors cause a suboptimal plan to be delivered to a patient. These errors may be reduced using a more accurate dose calculation algorithm (e.g.; MC) during treatment plan optimization, resulting in highly accurate dose distributions. A work done by Dogan et al. [25] showed that forty percent of the head and neck IMRT patients exhibited a convergence error of 5% in at least one DVH endpoint. Figure 5 shows that the MC-recalculated IMRT plans were less homogeneous. This might be due to the fact that the SC was used during the optimization. The homogeneity would have been improved if MC was used during the optimization.

The MC recomputed SIB prostate IMRT treatment plans showed that patient target doses were less than the one predicted by the treatment planning system's SC algorithm. For some cases, the MC recalculated doses were less than the SC calculated doses by nearly 2 Gy for all dose-volume indices. The deviations of this magnitude in target doses clearly indicate that the required target volume coverage by the protocol was not achieved for some patients and this may have clinical implications.

The MC dose verification tool presented in this work is fully integrated with our treatment planning system and showed itself to be a reliable tool to verify each of the clinical IMRT treatment plans. Furthermore, different from conventional dose verification methods, this tool allows assessment of dose distributions within the patient.

## Conclusion

MC dose calculation was used to recalculate dose distributions for twenty-two SIB-IMRT prostate plans that was originally optimized and calculated with SC. Measurements in phantom confirmed that MC agreed better with film measurements than the SC. Differences between the MC and SC computations in patient plans are likely to arise due to the errors in fluence prediction, photon leakage through patient, and photon transport through MLC leaves for SC based calculations. It is important to investigate the contribution of each error and determine the exact causes of these deviations between SC and MC calculated doses since this may have clinical implications. These results demonstrate that MC clearly can play an important role in SIB IMRT treatments. The results of this study and those of other treatment sites have resulted in the implementation of MC-based IMRT at our institution.

## Competing interests

The authors declare that they have no competing interests.

## Authors' contributions

All authors read and approved the final manuscript.

ND designed the study and performed the MC simulations and data analysis, and revised the manuscript. IM participated in data analysis. YW participated in data collection and revised the manuscript. PJK participated in design of the manuscript and revised the manuscript. JVS participated in design of the manuscript and revised the manuscript. MPH participated in data collection and revised the manuscript.
